# Leaner Women with Impaired Insulin Secretion Accounts for about 40% of Gestational Diabetes Mellitus in Japan

**DOI:** 10.1155/2019/7578403

**Published:** 2019-06-02

**Authors:** Seishi Furukawa, Yoichi Kobayashi

**Affiliations:** Department of Obstetrics & Gynecology, School of Medicine, Kyorin University, Tokyo, Japan

## Abstract

**Aim:**

To identify the involvement of leanness and impaired insulin secretion with Japanese gestational diabetes mellitus (GDM).

**Method:**

A cross-sectional study was conducted comprising 219 at-risk pregnant women who underwent a 75g glucose tolerance test at a single institute in Tokyo, Japan. We identified GDM and normal glucose tolerance (NGT). The cut-off value of the homeostasis model assessment insulin resistance (HOMA-IR) for detecting GDM was determined. The GDM group was divided into subgroups according to insulin resistance based on the cut-off value of HOMA-IR. We compared the prepregnancy body mass index (BMI) and homeostasis model assessment of *β*-cell function (HOMA-*β*) between the group comprising low insulin resistance (LIR) and the group comprising high insulin resistance (HIR).

**Results:**

Seventy GDM cases and 149 NGT cases were identified. By using receiver operating characteristic curve analysis, the HOMA-IR cut-off value was determined to be 1.41. Twenty-five GDM cases (36%) were classified as LIR and forty-five GDM cases (64%) were classified as HIR. The background including indications for having 75gOGTT and the gestational age having 75gOGTT did not differ between groups. The BMI of the LIR group was significantly lower than that of the HIR group (20.9±2.8 vs. 24.4 ± 5.5, p<0.01), and the HOMA-*β* of the LIR group was significantly lower than that of the HIR group (95.5±30.3 vs. 146.0±70.1, p<0.01). A positive linear correlation was found between BMI and HOMA-*β* in cases of GDM (r=0.27, p=0.02).

**Conclusion:**

Leanness with impaired insulin secretion is deeply involved in Japanese gestational diabetes mellitus.

## 1. Introduction

Normally, pregnant women produce sufficient amounts of insulin to overcome insulin demand and resistance which is caused by anti-insulin hormone produced from the placenta. However, in cases of women who have either more insulin resistance or impaired insulin secretion at pregnancy, gestational diabetes mellitus (GDM) then develops [1]. To date, studies have focused on investigating the increase in insulin resistance during pregnancy as a major cause of GDM [2, 3]. There is a dramatic increase in the prevalence of obesity in the world [4–6], and obesity can lead to a greater risk of GDM [7–10].

Looking at the current situation of obesity in Japan, the incidence of obesity in women over 20 years of age is 19%. By age group, obesity is the highest (23.8%) in women in their 70s and the lowest (6.6%) in women in their 30s. On the other hand, the percentage of leanness in women over 20 years of age is 12.4%. By age group, leanness is the highest (22.3%) in women in their 20s and the lowest (6.7%) in women in their 60s [11]. Therefore, at reproductive age, there is a tendency towards leanness in Japanese women.

In recent years, the problem of lean type 2 diabetes mellitus (type 2 DM) has become a focus of attention in that impaired insulin secretion is involved in the onset of type 2 DM [12]. There are fewer cases of obesity in Japanese compared to Caucasians [13, 14], and the insulin secretion ability in Japanese is significantly lower than that of Caucasians, which is strongly involved in the onset of type 2 DM [15]. It is thought that impaired insulin secretion in lean Japanese women may be strongly involved in the onset of GDM; however, only a few studies have reported on its involvement and frequency with respect to GDM [16–18].

GDM is a preliminary group of type 2 DM in the future. Other than being obese and having high insulin resistance, impaired insulin secretion in leanness may be involved in the onset of GDM and affected individuals who are lean prior to pregnancy may progress early on to GDM or worsen perinatal prognosis. We therefore conducted a cross-sectional study of at-risk pregnant women who underwent a 75g glucose tolerance test (75gOGTT) in an effort to evaluate the contribution of leanness with impaired insulin secretion in the onset of GDM. Our results provide sufficient data to assist in clarifying the mechanism leading to GDM in the Japanese population.

## 2. Methods

This study was undertaken retrospectively and approval (H27-117) was obtained from the constituted ethics committee of the Kyorin University. According to our ethics committee, retrospective research can be conducted by disclosing information on the bulletin board or on the website. The information includes ensuring that the subjects of the study have an opportunity to refuse to participate in the study, and if the patient refuses to participate in the study, it is excluded from the study. The medical charts of all women who underwent 75gOGTT due to perceived risk of GDM at Kyorin University from April 2013 to May 2015 were retrospectively examined. Kyorin University is a tertiary center located in a suburb of Tokyo.

During the study period, at the first visit of pregnancy and midpregnancy (24~28 weeks of gestation), measurement of random plasma blood glucose levels was performed for all pregnant women. In the plasma blood glucose method, 100mg/dL or more was regarded as positive for screening and 75gOGTT was then performed. For pregnant women who were not diagnosed with GDM or clinical diagnosis of diabetes mellitus at the first visit, women had the opportunity of being screened for GDM at midpregnancy. Thus, routine screening for GDM was performed in two stages. Aside from routine screening, if pregnant women had any risk factors for GDM including obesity, history of GDM, GDM-associated adverse pregnancy outcomes (miscarriages, stillbirths, and intrauterine fetal death), family history of DM in the first-degree relative, polyhydramnios in the current pregnancy, fetus as heavy for date in the current pregnancy, and repeated positive results in the urine glucose test strip, these women also underwent 75gOGTT at the discretion of the attending physician. Pregnant women who were screened positive and who underwent 75gOGTT served as part of the study group.

GDM is diagnosed if one or more of the following criteria is met in a 75gOGTT during pregnancy: (1) fasting plasma glucose level ≥92 mg/dL (5.1 mmol/L), (2) 1 h value ≥180 mg/dL (10.0 mmol/L), and (3) 2 h value ≥153 mg/dL (8.5 mmol/L) according to diagnostic criteria of diabetes mellitus in Japan [19]. Overt diabetes in pregnancy is diagnosed if either fasting plasma glucose level is ≥126 mg/dL (7.0 mmol/L) or HbA1c is ≥6.5% [19]. GDM cases and normal glucose tolerance (NGT) cases were included in this study.

We firstly determined the homeostasis model assessment insulin resistance (HOMA-IR) cut-off value for detection in women with GDM from the study group. HOMA-IR was calculated as follows: fasting insulin (*μ*U/ml)×fasting plasma glucose (mg/dl)/405. Insulin was measured with a chemiluminescent automated method (CLIA) (ABBOT JAPAN CO., LTD.). The cross-reaction with proinsulin is less than 0.1%. GDM women were then divided into subgroups according to insulin resistance based on the HOMA-IR cut-off value. GDM women with the HOMA-IR cut-off value or greater were designated as the group comprising high insulin resistance (HIR). GDM women with less than the cut-off value were designated as the group comprising low insulin resistance (LIR).

The following maternal demographic data of the LIR and HIR groups were collected: maternal age, parity (primipara), prepregnancy BMI, random plasma blood glucose at the first visit, indication for having 75gOGTT, and gestational age at undergoing 75gOGTT. The homeostasis model assessment of *β*-cell function (HOMA-*β*) was used as an insulin secretion index. HOMA-*β* was calculated as follows: 360×fasting insulin (*μ*U/ml)/[fasting plasma glucose (mg/dl) – 63]. We compared the plasma glucose concentrations following 75gOGTT, the prepregnancy body mass index (BMI), and HOMA-*β* between LIR and HIR groups. We also examined the relationship between prepregnancy BMI and HOMA-*β* in GDM women.

Neonatal data of birth weight (g) were corrected. The percentage of heavy for date (HFD) was compared between the LIR and HIR groups. Heavy for date (HFD) was defined as sex-specific birth weight more than the 90th percentile for gestational age according to the Japanese standard growth curve for singleton.

Data are expressed as number, incidence (%), median, range, interquartile range (IQR), or mean ± SD. Receiver operating characteristic (ROC) curve analysis was used to determine the HOMA-IR cut-off value in the study group. Comparisons between groups were made using Welch's t test, *χ*^2^ test, or Spearman's correlation coefficient. Comparison among groups was made using the one-way ANOVA. Post hoc analysis was performed using the Bonferroni-Dunn test. Probability values < 0.05 were considered significant.

## 3. Results

**Figure 1:**
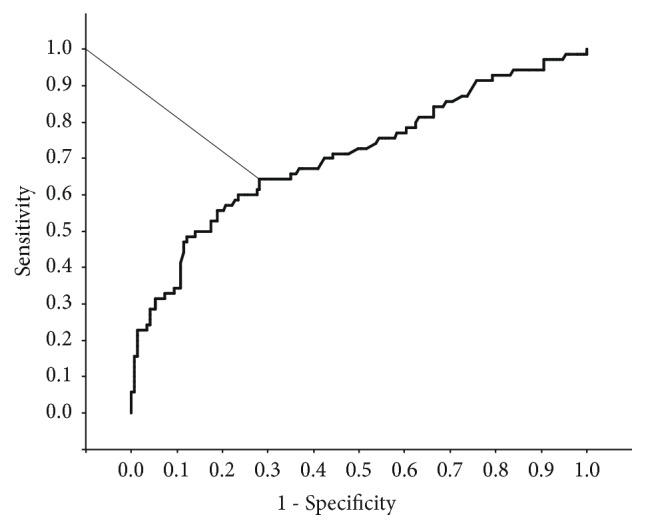
ROC curve of HOMA-IR for distinguishing GDM cases from the study group. HOMA-IR: homeostasis model assessment insulin resistance. GDM: gestational diabetes mellitus.

During the study period, 219 at-risk pregnant women who underwent 75gOGTT served as the study group. Seventy GDM women and 149 NGT women were identified from the study group. There were no cases of overt diabetes in pregnancy during study period. As seen from the ROC curve in Figure 1, the area under the curve was 0.71 with statistical significance (p<0.01), and the shortest distance from the upper left corner and the maximum product of sensitivity and specificity was at 1.41 (sensitivity 0.64, specificity 0.72). The HOMA-IR cut-off value was therefore determined to be 1.41.

GDM women were then divided into subgroups according to insulin resistance based on the HOMA-IR cut-off value of 1.41. Eventually, 25 GDM women (35.7%) were classified as LIR and 45 GDM women (64.2%) were classified as HIR.

Age, percentage of primipara, and random plasma blood glucose at the first visit did not differ significantly between LIR and HIL groups. Indications for having 75gOGTT and the gestational age having 75gOGTT also did not differ between LIR and HIL groups (Table 1).

**Figure 2:**
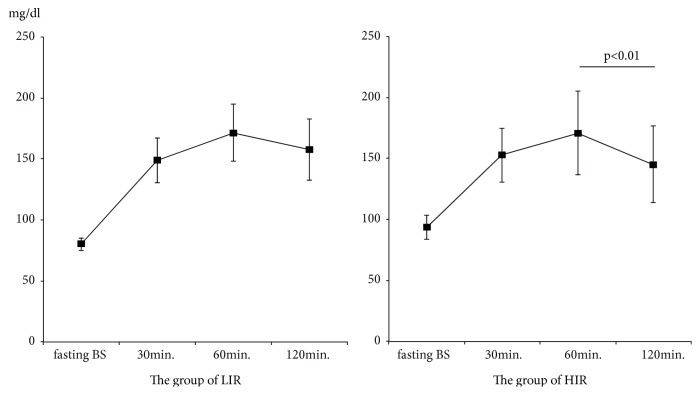
Glucose serum concentrations following 75gOGTT in the LIR and HIR groups. LIR group: women with low insulin resistance, HIR group: women with high insulin resistance. Comparison was made using the one-way ANOVA. Post hoc analysis was performed using the Bonferroni-Dunn test. Data are expressed as mean ± SD. The value of glucose did not differ significantly at 60 and 120 minutes in the LIR group. The value of glucose at 120 minutes was significantly lower than that at 60 minutes in the HIR group.

**Figure 3:**
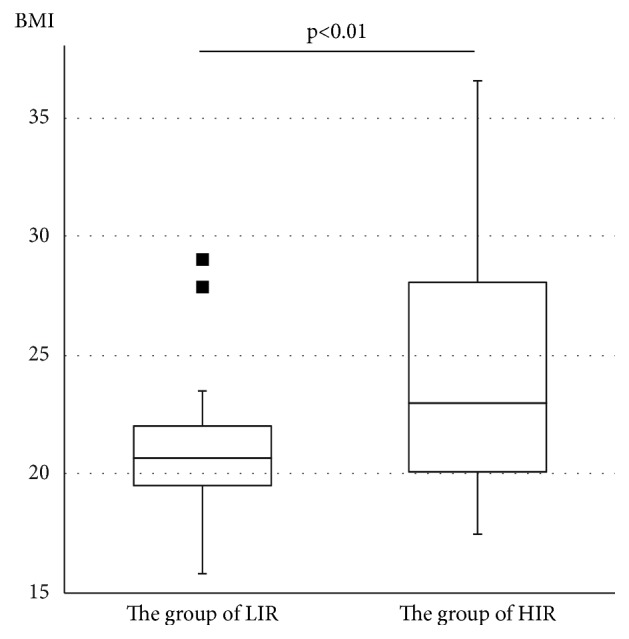
Comparison of BMI between the LIR and HIL groups. LIR group: women with low insulin resistance, HIR group: women with high insulin resistance. Comparison between groups was made using Welch's t test. The black bar represents the median. The interquartile range is represented by a box. The BMI of the LIR group is significantly lower than that of the HIR group. The value exceeding 1.5 IQR was represented by closed square.

**Figure 4:**
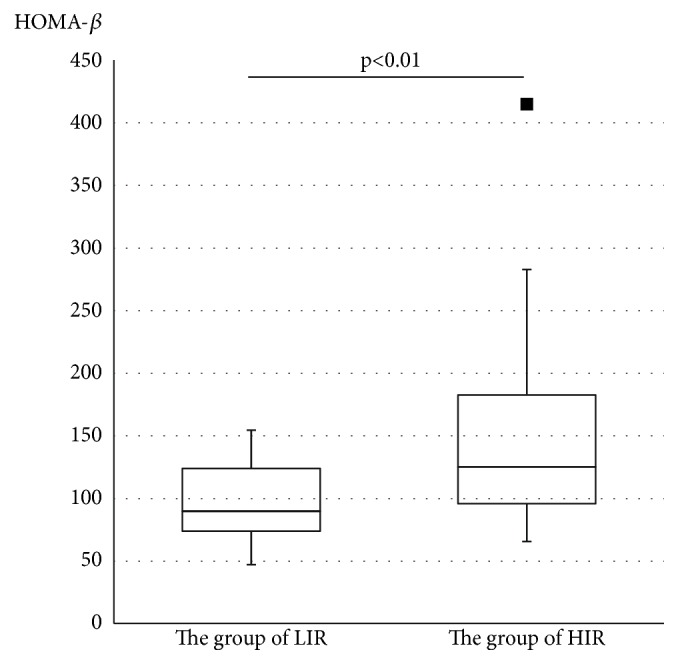
Comparison of HOMA-*β* between the LIR and HIL groups. LIR group: women with low insulin resistance, HIR group: women with high insulin resistance. HOMA-*β*: Homeostasis model assessment of *β*-cell function. Comparison between groups was made using Welch's t test. The black bar represents the median. The interquartile range is represented by a box. Values exceeding 1.5 IQR were represented by closed squares. The HOMA-*β* of the LIR group is significantly lower than that of the HIR group.

**Figure 5:**
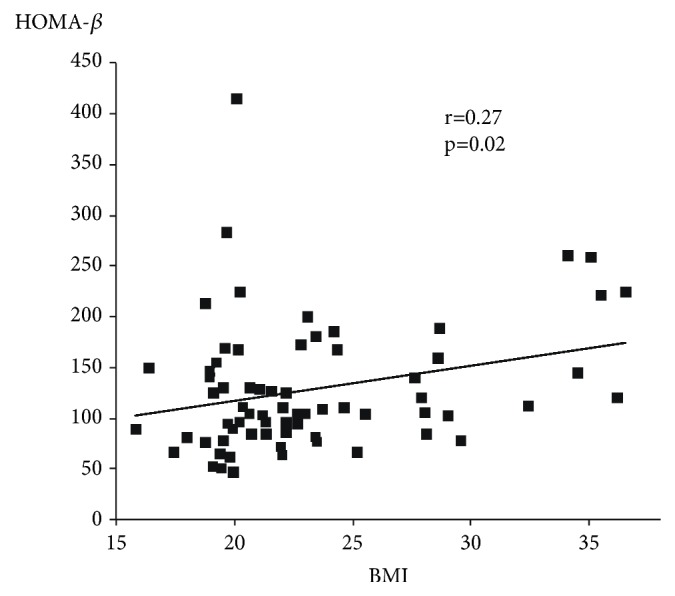
The relationship between prepregnancy BMI and HOMA-*β* in GDM women. HOMA-*β*: Homeostasis model assessment of *β*-cell function, GDM: gestational diabetes mellitus. A positive linear correlation was found between the BMI and HOMA-*β* with respect to GDM.

Following 75gOGTT, the value of plasma glucose at 60 minutes was the highest in both LIR and HIR groups. The value of plasma glucose at 120 minutes in the LIR group remained high, and the value of plasma glucose did not differ significantly at 60 and 120 minutes (p=0.09, Figure 2). On the other hand, in the HIL group, the value of plasma glucose at 120 minutes was significantly lower than that at 60 minutes (p<0.01, Figure 2). The BMI of the LIR group was significantly lower than that of the HIR group (20.9±5.5 vs. 24.4±5.5, p<0.01, Figure 3), and HOMA-*β* of the LIR group was significantly lower than that of the HIR group (95.5±30.3 vs. 146.0±70.1, p<0.01, Figure 4). A positive linear correlation was found between the BMI and HOMA-*β* with respect to GDM (r=0.27, p=0.02, Figure 5).

The absolute birth weight did not differ significantly between LIR and HIL groups (2950.3±375.0g vs. 2805.3±743.8g, p=0.28). However, the percentage of HFD in the LIR group was significantly lower than that of the HIR group (4% vs. 27%, p=0.02).

## 4. Discussion

In this study, about 40% of GDM women (LIR group) were without increases in insulin resistance. Moreover, GDM women without increases in insulin resistance also had impaired insulin secretion and tended to be leaner prior to pregnancy compared to GDM women with high insulin resistance (HIR group). When looking at changes in plasma glucose concentrations following 75gOGTT, values did not decrease by as much at 120 minutes in the LIR group (Figure 2). In contrast, values at 120 minutes showed a marked decline in the HIR group. The persistence of high plasma glucose in the LIR group is considered to reflect impaired insulin secretion. Thus, there are two groups having obviously different causes for the development of GDM in the Japanese population, and the proportion of GDM without increases in insulin resistance is relatively high.

The incidence of obesity in the Japanese population is relatively low [13, 14], and obesity is known to be linked to increased insulin resistance. In fact, Japanese have a lower insulin secretion ability at the stage of normal glucose tolerance compared to Caucasians. Caucasians show a sharp increase in insulin resistance as type 2 diabetes mellitus (type 2 DM) develops. In contrast, when Japanese become more diabetic, insulin secretion ability is more impaired than the increase in insulin resistance [15]. Thus, impaired insulin secretion is more important for the onset of type 2 DM in the Japanese population. In a report on impaired insulin secretion regarding Japanese GDM, impaired insulin secretion was noted in the measurement at the puerperium period [20, 21]. The presence of GDM due to impaired insulin secretion, apart from GDM due to elevated insulin resistance during pregnancy, has been recently reported [22]. We also revealed that GDM women without increases in insulin resistance had impaired insulin secretion at midpregnancy. Those data suggest that impaired glucose tolerance caused by impaired insulin secretion confirmed in Japanese with type 2 DM also occurs in Japanese GDM.

Generally, prognosis during pregnancy worsened as obesity developed. In fact, pregnancy complications are increasing in the Japanese population if prepregnancy BMI based on 2009 criteria recommended by the Institute of Medicine is high [23]. In this study, we showed that GDM women without increases in insulin resistance tended to be more lean (mean BMI: 20.9) before pregnancy compared to GDM women with high insulin resistance (mean BMI: 24.4, Figure 3). Relatively little is known about the worse prognosis of prepregnancy leanness on pregnancy; however, it is necessary to keep in mind that it may increase GDM in Japanese women. It is also known that Asians are relatively lean and insulin sensitivity is high with low insulin secretion ability [24]. Furthermore, in Japanese lean women, there is a risk of a decrease in the “amount” of muscle tissue, and fat accumulation in muscle tissue causes insulin resistance followed by developing DM [25]. We need to survey the maternal and fetal prognosis of leanness with both endocrinological predisposition by race and environmental factors given that there are populations prone to GDM.

Under such circumstances, the percentage of HFD in the LIR group was significantly lower than in the HIR group in this study. There is no difference in the timing of diagnosis of GDM and they are similarly subject to intervention after they are diagnosed with GDM. We previously reported that HOMA-IR shows a significantly positive correlation with placental and birth weights and increased insulin resistance has a negative effect on placental efficacy [26]. Consequently, insulin resistance might be an independent factor in determining fetal weight gain. Having fewer infants with HFD in the LIR group may be advantageous for perinatal prognosis. Nevertheless, it has resulted in the distinctive clinical features during perinatal period of each group.

There were several limitations in our study. Firstly, although we decided that the HOMA-IR cut-off value from the study group included NGT, our NGT is also originally a population comprising certain factors. It may not be appropriate to include our NGT in a group that is employed to determine the cut-off value. In addition, we have not conducted studies on insulin secretion and resistance in our NGT. Comparison with NGT may make the involvement of insulin secretion failure and insulin resistance in GDM clearer. Secondly, although insulin resistance and secretory ability were evaluated by HOMA-IR and HOMA-*β*, those are simple evaluation methods and it cannot always be said that those are accurate evaluation methods. Since insulin secretion is deeply involved in changes in insulin sensitivity during pregnancy, it is necessary to test using more accurate tests like glucose clamp technique. As a third, we did not evaluate any indicators for perinatal prognosis other than birth weight between the two groups. Further examinations are necessary to evaluate the difference between GDM with high insulin resistance and that without.

In conclusion, there are two groups having obviously different causes for the development of GDM in the Japanese population, and the proportion of leaner GDM women who have impaired insulin secretion and no increase in insulin resistance is relatively high. GDM is a preliminary group of type 2 DM in the future, and affected individuals who are lean prior to pregnancy are predicted to progress early on to type 2 DM. Further examinations including insulin resistance and insulin secretion ability during pregnancy and efforts to understand the cause of GDM will ensure the future health of pregnant women.

## Figures and Tables

**Table 1:** Maternal characteristics of the LIR and HIR groups.

Number	HIR	LIR	p
	45	25	
maternal age (yrs)	36.0±4.6	36.8±4.6	0.76
primipara (%)	24 (53)	17 (68)	0.23
random blood glucose at the first visit (mg/dl)	91.5±12.6	95.0±20.7	0.46
gestational age at undergoing 75gOGTT (wks)	24.6±6.2	21.7±6.7	0.08

Indication for 75gOGTT			
random blood glucose method, 100mg/dL or more at first visit	12	3	0.48
random blood glucose method, 100mg/dL or more at mid-pregnancy	16	8	
repeated positive results in the urine glucose test strip	10	11	
history of GDM	1	1	
obese	1	0	
others	5	2	

LIR group: women with low insulin resistance, HIR group: women with high insulin resistance, 75gOGTT: 75g glucose tolerance test, GDM: gestational diabetes mellitus

## Data Availability

The data used to support the findings of this study are available from the corresponding author upon request. The datasets generated during and/or analyzed during the current study are not publicly available due to protection of personal information but are available from the corresponding author on reasonable request.
